# A case of HDR syndrome with recurrent matured ovarian teratomas

**DOI:** 10.1530/EDM-24-0140

**Published:** 2025-09-11

**Authors:** Ryizan Nizar, Louise Sarr, Tim Saunders, Waleed Elsayed, Arjun Joshi

**Affiliations:** Great Western Hospital, Swindon, UK

**Keywords:** calcium, HDRS, hypoparathyroid, rare diseases/syndromes, ovarian teratoma

## Abstract

**Summary:**

HDR syndrome is a rare, heterogeneous genetic disorder characterised by a triad of hypoparathyroidism, sensorineural deafness, and renal disease. The defect in most patients is caused by deletions in chromosome 10p14 or mutations in the *GATA3* gene. HDR syndrome is also associated with several atypical features, including eye, skin, neurological, cardiac, gastrointestinal, and urogenital involvement. We report the case of a 27-year-old Caucasian woman with HDR syndrome (*GATA3* NM_001002295.1: c.977C>A p. (Thr326Asn)), who presents with multiple atypical associated features. She has also had recurrent benign ovarian cystic teratomas, although it is unclear whether these are related to HDR syndrome, as this has never been reported.

**Learning points:**

- HDR syndrome is a rare autosomal genetic disorder characterised by a triad of hypoparathyroidism, sensorineural deafness, and renal disease.
- It may be associated with atypical features involving various organs, which will require investigation and management.
- There is a paucity of evidence and guidance on managing hypocalcaemia in HDR syndrome. Given that HDR syndrome causes agenesis rather than a receptor issue, calcium levels should be targeted between 2.0 and 2.2 mmol/L, similar to the management of hypoparathyroidism caused by surgery or autoimmune disease.

## Background

HDR syndrome is a rare, heterogeneous genetic disorder characterised by a triad of hypoparathyroidism, sensorineural deafness, and renal disease. In most patients, the defect is caused by deletions on chromosome 10p14 or mutations in the *GATA3* gene (1). These genetic defects disrupt the normal development of the parathyroid glands, inner ear, and kidneys, resulting in the hallmark features of the syndrome.

The exact prevalence of HDR syndrome remains unknown, with about 180 cases reported worldwide in the literature (2). So far, 93 *GATA3* pathogenic variants have been reported in the literature (1).

A *GATA3* analysis and phenotypic spectrum described in nine Japanese families with HDR syndrome by Muroya *et al.* identified heterozygous *GATA3* abnormalities in seven of the nine families. These were associated with a wide phenotypic spectrum, with components of the HDR syndrome variably manifested (3).

HDR syndrome is also associated with atypical features, including retinitis pigmentosa, atrophy of the retinal pigment epithelium (4), psoriasis, recurrent cerebral infarctions in the basal ganglia (5), basal ganglia calcifications, congenital cardiac abnormalities, pyloric stenosis, Hirschsprung’s disease, growth failure, female genital tract abnormalities, and cognitive disability.

While there are no specific treatment guidelines exclusively for HDR syndrome, management is based on established clinical practices for treating the core symptoms (hypoparathyroidism, sensorineural deafness, and renal anomalies) and atypical features. Multidisciplinary care is essential to optimise outcomes, as highlighted in our case.

The condition is rare, and its prognosis is influenced by the specific features of the syndrome in each patient, such as the severity of hypoparathyroidism, sensorineural deafness, renal agenesis, and the presence of atypical features.

We report a case of a 27-year-old Caucasian woman with HDR syndrome (*GATA3* NM_001002295.1: c.977C>A p. *(Thr326Asn)*), with multiple atypical associated features.

## Case presentation

Our patient was diagnosed with congenital hypoparathyroidism in childhood but was lost to follow-up. She was initially admitted to our centre with muscle cramps and paraesthesia in 2020 at the age of 25 years with a low serum calcium of 1.85 mmol, parathyroid hormone of 1.1 pmol/L, and magnesium of 0.69 mmol/L (Table 1). Clinical symptoms, calcium levels, and management before 2020 are unknown, as these were undertaken at different hospitals.

**Table 1:** Results of investigations.

Investigation	Result	Reference
Calcium, mmol/L	1.85	2.2–2.65
Phosphate, mmol/L	1.92	0.81–1.45
Vitamin D, nmol/L	31	32–50
Parathyroid hormone, pmol/L	1.1	1.3–9.3
Creatinine, μmol/L	98	45–84
Magnesium, mmol/L	0.69	0.7–1

She experienced several hospital admissions for symptomatic hypocalcaemia over the following year. She has right-sided unilateral renal agenesis and a hypertrophic left kidney. She also has sensorineural hearing loss, with 70% loss in the left ear and 30% loss in the right.

In addition, she exhibits several known atypical features associated with HDR syndrome.

### Eyes

She is partially sighted. Although there was no evidence of retinitis pigmentosa, which is usually associated with HDR, she has bilateral retinal thinning, which has also been reported in association with HDR syndrome (4). Her optical coherence tomography revealed thin retinal tissue at the maculae with normal layering and an adherent posterior vitreous face. She also has left-sided exotropia, congenital nystagmus, amblyopia, and bilateral hypermetropia.

### Skin

She was diagnosed with psoriasis during childhood. The association between psoriasis and HDR syndrome has been documented. Hypocalcaemia due to hypoparathyroidism affects cell adhesion molecules such as cadherins, which require calcium. Psoriasis symptoms tend to improve once calcium levels are normalised (5).

### Brain

**Figure 1:**
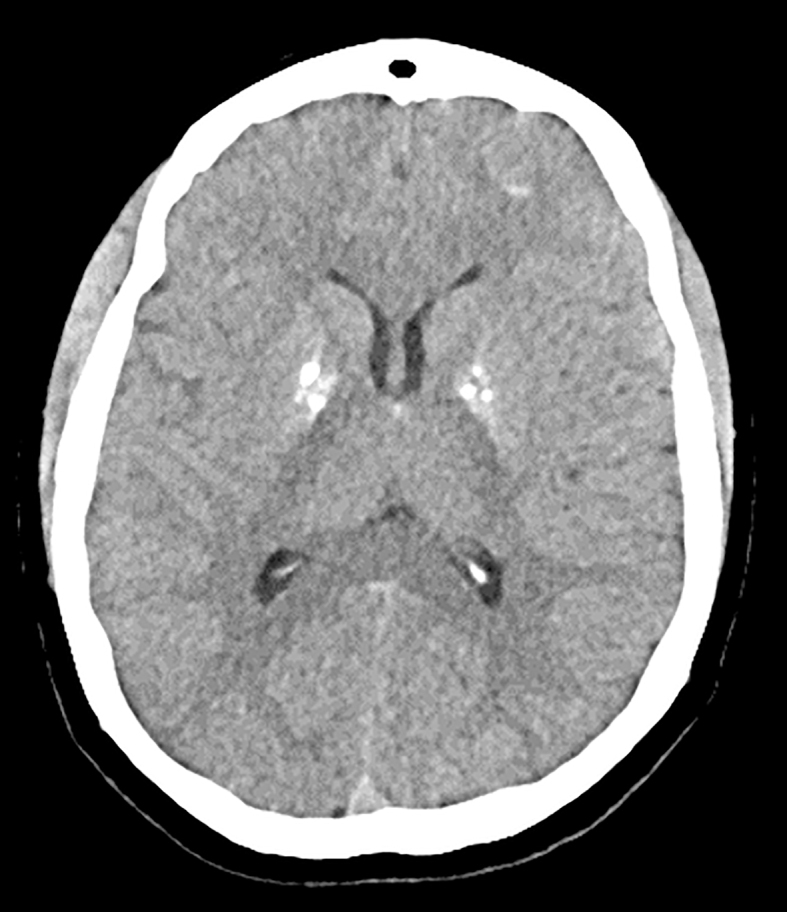
An axial non-contrast CT brain that shows bilateral punctate calcification of the globus palladi.

A CT head scan in 2016 showed basal ganglia calcifications, indicative of hypoparathyroidism with associated hypocalcaemia and hyperphosphataemia (Fig. 1). Hypoparathyroidism is known to cause ectopic or metastatic calcium deposition in soft tissues, including the brain, kidneys, lungs, and stomach.

### Ovaries

**Figure 2:**
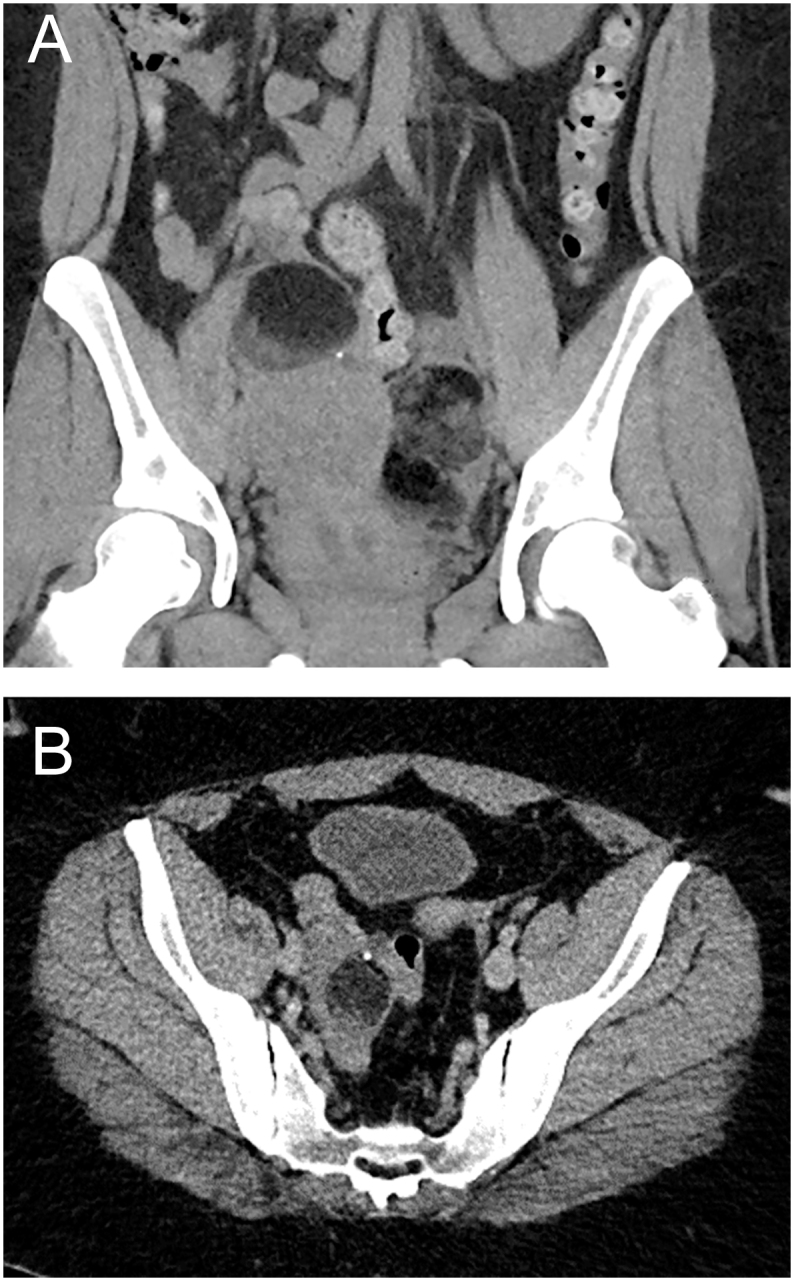
(A) Coronal post-contrast CT abdomen/pelvis showing bilateral ovarian masses containing both soft tissue and fat, in keeping with an ovarian mature cystic teratoma. (B) Left ovarian mass containing soft tissue, fat, and calcification. Soft tissue could represent a Rokitansky nodule within the ovarian MCT.

**Figure 3:**
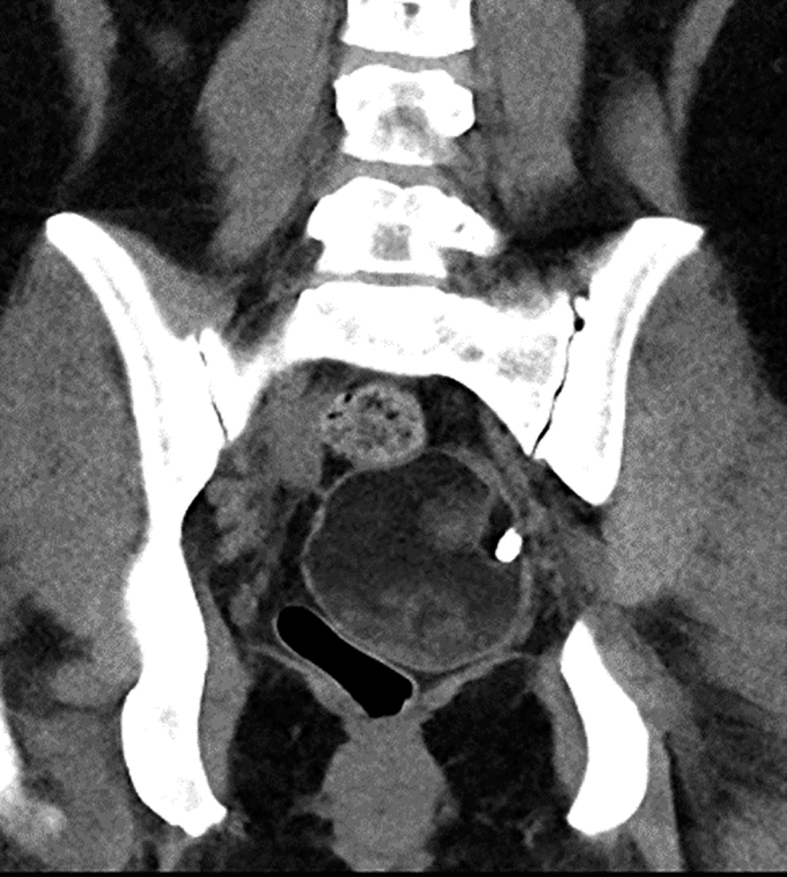
Post-operative scan showing recurrence of the teratoma within the right ovary.

In 2021, she presented with lower abdominal pain. A CT scan of the urinary tract revealed large bilateral ovarian dermoid cysts of mixed attenuation, primarily composed of fat, with a vestigial tooth in the left cyst (Fig. 2). The left ovarian dermoid measured 10 × 8 × 7 cm and the right measured 6 × 6 × 5 cm. She underwent a laparoscopic bilateral cystectomy, with histology confirming mature cystic teratomas (MCTs) (Table 2). In 2023, she underwent surgery again to remove a recurrent teratoma in the ovary (Fig. 3).

**Table 2:** Histology results.

	Macroscopic	Microscopic
1	Left ovarian dermoid cyst. Multiple fragmented pieces of cyst wall with yellow cheesy material and hair, altogether weighing 41 g and measuring 100 × 55 × 55 mm in aggregate. Fragments of a predominantly smooth internal surface with some more solid areas, and some calcification/opacification noted	MCT. The elements included are stratified squamous epithelium, skin appendages, adipose tissue, and respiratory epithelium. There is a large amount of unremarkable background ovarian stroma included
2	Right ovarian dermoid cyst. Specimen consists of multiple fragments of cyst wall with hair and cheesy material, altogether weighing 18 g and measuring 65 × 40 × 15 mm in aggregate	Mature teratoma with virtually only attenuated squamous epithelium as the lining of the cyst. In some areas, there is a reaction to hair particles, suggesting rupture of the cyst contents into the wall of the cyst
		Summary: Bilateral ovarian MCTs

MCTs, mature cystic teratomas.

In late 2023, she experienced a further recurrence. The case was extensively discussed at the gynaecology multidisciplinary team meeting. It was also noted that she had extensive endometriosis. In view of the recurrent ovarian teratomas, she underwent a left oophorectomy and is under close ongoing surveillance. If there is further recurrence on the right, the plan is to offer a right oophorectomy as well.

Although HDR syndrome has been associated with several female genital tract malformations (6), to our knowledge, there have been no previous reports of ovarian cystic teratomas in patients with HDR syndrome in the literature.

### Gastrointestinal system

Our patient has also struggled with episodic recurrent vomiting for several years. Although Hirschsprung’s disease is associated with HDR syndrome (7), her age at presentation argues against this diagnosis. However, although rare, Hirschsprung’s disease can be diagnosed in adulthood. She has been evaluated by the gastroenterology team, and the diagnosis of Hirschsprung’s disease has been excluded.

She has no family history of hypocalcaemia or hypoparathyroidism other than her maternal grandmother, who reportedly suffered from hypoparathyroidism, although we have not been able to verify this. Her parents were referred to the local Clinical Genetics team for family screening. Genetic testing returned negative results, suggesting this is a *de novo* presentation.

## Investigation

Genetic tests confirmed a *GATA3* mutation Chr10: g.8111488 with variant NM_001002295.1: c.977C>A p.

## Treatment

At presentation, our patient remained symptomatic for hypocalcaemia despite 1,600 mg elemental calcium per day. She was admitted for IV calcium replacement, and her subsequent oral calcium was switched to an effervescent preparation, as she struggled with tablets. In addition, she was started on alfacalcidol with a dose up to 3 μg once daily and vitamin D. Despite these adjustments, we continued to face difficulty achieving the desired serum calcium targets, leading to the decision to switch her to calcitriol to better control calcium levels and relieve her symptoms.

Eventually, the patient was started on daily teriparatide injections, which initially stabilised her calcium levels. Unfortunately, after a few weeks, she experienced recurrent hypocalcaemia. At that point, we switched to a continuous parathyroid hormone (PTH) infusion using a Medtronic 740 Pump. This approach helped significantly for a few months; however, she again developed hypocalcaemia, which required intermittent IV calcium.

Currently, the patient is being managed with a high dose of calcitriol, calcium tablets, and a teriparatide infusion via an insulin pump. Despite this, she still receives about two infusions of calcium in an ambulatory care setting. In addition, she has been referred to the local endocrine surgical centre for consideration of a parathyroid transplant.

The patient is under the care of the dermatology team for her psoriasis and continues to receive follow-up and surveillance from the gynaecology team for the recurrence of ovarian teratomas. She is also being investigated by the gastroenterology team for her recurrent vomiting.

Furthermore, she is under follow-up by the ENT team for her sensorineural deafness and is awaiting surgery for a hearing aid implant.

## Outcome and follow-up

Our patient is currently under the care of multiple specialties. Management of her hypocalcaemia remains challenging.

She is being considered for a hearing implant and also continues to have close follow-up with the gynaecology team.

This 27-year-old woman presented with all three components of the HDR triad: hypoparathyroidism (H), bilateral sensorineural deafness (D), and right renal agenesis (R). She also has psoriasis, basal ganglia calcification, and visual impairment with retinal thinning, all of which are associated with HDR syndrome.

In addition, she has bilateral ovarian teratomas with recurrence, which, to our knowledge, has not been previously reported in the context of HDR syndrome.

The defect in most patients with HDR syndrome is caused by deletions in chromosome 10p14 or mutations in the *GATA3* gene. This results in haploinsufficiency of the dual zinc finger transcription factor *GATA3* on the short arm of chromosome 10p. *GATA3* encodes a transcriptional regulator essential for the development of the parathyroid glands, inner ear, kidneys, and thymus (8).

The prevalence of HDR syndrome is equal across ethnic groups, genders, and ages of diagnosis (1).

Hypoparathyroidism occurs in approximately 93% of patients, deafness in about 96%, and renal disease in about 72% (1). The likelihood of occurrence of each component increases with age, and by age 50, all patients will likely have all three components of the syndrome (1).

The goals of therapy in chronic hypoparathyroidism are to relieve symptoms, raise and maintain the serum calcium concentration just below the normal range (e.g. 8.0–8.5 mg/dL (2.0–2.1 mmol/L)), and prevent the iatrogenic development of kidney stones. Higher calcium values are not necessary and are typically limited by the development of hypercalciuria due to the loss of renal calcium-retaining effects of PTH (9).

There is limited evidence or guidance on managing hypocalcaemia in HDR syndrome. Given that HDR syndrome causes agenesis rather than a receptor issue, we aimed to target our patient’s calcium levels between 2.0 and 2.2 mmol/L, similar to hypoparathyroidism caused by surgery or autoimmune disease (10)

Management of kidney disease depends on the nature of the abnormality, and prognosis mainly depends on the severity of the disease (1). Sensorineural deafness can be managed with hearing amplification and cochlear implantation.

Genetic counselling is a fundamental part of managing HDR syndrome (1), and our patient and her first-degree relatives were referred to the Clinical Genetics team.

MCT of the ovary is the most common type of ovarian germ cell neoplasm, occurring in approximately 20% of all ovarian neoplasms. These tumours arise from a single germ cell after the first meiotic division (11). Caspi *et al.* suggested a possible genetic factor in the pathogenesis of these tumours, with 9.8% of the study group found to have at least one first-degree relative with dermoid cysts or teratomas, compared to only 2% in the control group.

With regard to any potential association between HDR syndrome and ovarian teratomas, *GATA3* protein is also considered a valuable marker for certain types of urinary bladder and urethral cancers, as well as for parathyroid gland tumours.

We acknowledge that there is no certainty that the ovarian teratomas in this patient are related to the *GATA3* variant or HDR syndrome, as this is a single case.

## Declaration of interest

The authors declare that there is no conflict of interest that could be perceived as prejudicing the impartiality of the research reported.

## Funding

This work did not receive any specific grant from any funding agency in the public, commercial, or not-for-profit sector.

## Patient consent

R N discussed publication of this case directly. Written signed consent was gained from the patient for publication of the submitted article and accompanying images.

## Author contribution statement

RN provided direct clinical care and wrote the manuscript. LS assisted in providing clinical care and manuscript writing; TS reported and provided descriptions of the CT head and abdominopelvic CT images; WE managed the gynaecology aspects of the case and the manuscript; AJ wrote the manuscript.
